# Altered Regulation of KIAA0566, and Katanin Signaling Expression in the Locus Coeruleus With Neurofibrillary Tangle Pathology

**DOI:** 10.3389/fncel.2018.00131

**Published:** 2018-05-17

**Authors:** Pol Andrés-Benito, Raul Delgado-Morales, Isidro Ferrer

**Affiliations:** ^1^Neuropathology, Pathologic Anatomy Service, Bellvitge Biomedical Research Institute, Hospitalet de Llobregat, Bellvitge University Hospital, Barcelona, Spain; ^2^Cancer Epigenetics Group, Cancer Epigenetics and Biology Program, Bellvitge Biomedical Research Institute, L'Hospitalet de Llobregat, Spain; ^3^Department of Pathology and Experimental Therapeutics, University of Barcelona, L'Hospitalet de Llobregat, Spain; ^4^Institute of Neurosciences, University of Barcelona, L'Hospitalet de Llobregat, Spain; ^5^Biomedical Network Research Centre of Neurodegenerative Diseases, National Institute of Health Carlos III, L'Hospitalet de Llobregat, Spain

**Keywords:** Alzheimer's disease, locus coeruleus, neurofibrillary tangles, methylation, katanin, KIAA0556, microtubules

## Abstract

The locus coeruleus (LC), which contains the largest group of noradrenergic neurons in the central nervous system innervating the telencephalon, is an early and constantly vulnerable region to neurofibrillary tangle (NFT) pathology in aging and Alzheimer's disease (AD). The present study using whole genome bisulfite sequencing and Infinium Human Methylation 450 BeadChip was designed to learn about DNA methylation profiles in LC with age and NFT pathology. This method identified decreased DNA methylation of Katanin-Interacting Protein gene (*KIAA0566)* linked to age and presence of NFT pathology. *KIAA0566* mRNA expression demonstrated with RT-qPCR significantly decreased in cases with NFT pathology. Importantly, KIAA0566 immunoreactivity was significantly decreased only in LC neurons with NFTs, but not in neurons without tau pathology when compared with neurons of middle-aged individuals. These changes were accompanied by a similar pattern of selective p80-katanin reduced protein expression in neurons with NFTs. In contrast, p60-katanin subunit expression levels in the neuropil were similar in MA cases and cases with NFT pathology. Since katanin is a major microtubule-severing protein and KIAA0566 binds and interacts with katanin, de-regulation of the katanin-signaling pathway may have implications in the regulation of microtubule homeostasis in LC neurons with NFTs, thereby potentially interfering with maintenance of the cytoskeleton and transport.

## Introduction

Studies of Alzheimer's disease (AD) are mainly focused on the entorhinal cortex, hippocampus, and neocortex because of the massive accumulation with disease progression of β-amyloid deposits (diffuse and senile plaques) and hyper-phosphorylated tau-containing neurofibrillary tangles (NFTs), neuropil threads, and dystrophic neurites in these regions. Recently, the study of the brainstem in AD has captivated attention as (a) several nuclei of the brain stem such as the raphe nuclei and locus ceruleus (LC) are early and constantly affected in AD by abnormal neuronal deposition and formation of NFTs; (b) these nuclei are the main source of serotoninergic and noradrenergic innervations of the telencephalon including cerebral cortex; and (c) several functions dependent on the integrity of these nuclei such as arousal, attention, sleep-awake cycles, emotional states (control of panic, anxiety, and depression), autonomic function, memory and learning, stress responses, and motor coordination, among others, are altered in AD (Rüb et al., 2001; Aston-Jones and Cohen, 2005; Grinberg et al., 2009; Simic et al., 2009; Šimić et al., 2016; Attems et al., 2012; Szabadi, 2013). Whole-transcriptome arrays in LC reveal up-regulation of genes coding for proteins associated with heat shock protein binding and genes associated with ATP metabolism, and down-regulation of genes coding for DNA-binding proteins and members of the small nucleolar RNA family in LC neurons at early stages of NFT pathology (Andrés-Benito et al., 2017). MicroRNA expression is altered in the LC at early stages of NFT pathology (Llorens et al., 2017). These observations indicate alterations in the mechanisms leading to gene transcription and protein translation in LC at early stages of AD-related pathology.

DNA methylation and other epigenetic mechanisms which are modulators of gene transcription are altered in several neurodegenerative diseases including AD (Jakovcevski and Akbarian, 2012; Lu et al., 2013; Sanchez-Mut et al., 2014; Lardenoije et al., 2015; Blanch et al., 2016; Watson et al., 2016; Wen et al., 2016; Nicolia et al., 2017; Roubroeks et al., 2017; Smith and Lunnon, 2017). Epigenetic deregulation of brainstem nuclei has been postulated as one of the primary mechanisms in the pathogenesis of AD (Iatrou et al., 2017). The present work focuses on the study of DNA methylation in dissected LC in cases with NFT pathology compared with middle-aged individuals. Since one of the differentially methylated genes is *KIAA0556*, which encodes katanin-Interacting Protein, the study is then redirected to the analysis of expression of KIAA0556 mRNA and protein, and the proteins katanin subunit 60 and katanin subunit 80 (p60-katanin and p80-katanin, respectively) to assess possible alterations in katanin pathway signaling linked to NFT pathology in LC.

## Materials and methods

### Human brain samples

Human brain samples were obtained from the Institute of Neuropathology Brain Bank (HUB-ICO-IDIBELL Biobank) following the guidelines of the Spanish legislation on this matter (Real Decreto 1716/2011) and approval by the local ethics committee of the Bellvitge University Hospital-IDIBELL. The post-mortem interval between death and tissue processing was between 2 h 45 min and 15 h. This interval permits the study of RNA and protein expression as assessed elsewhere (Ferrer et al., 2008). One hemisphere was immediately cut in coronal sections, 1 cm thick, and selected areas of the encephalon were rapidly dissected, frozen on metal plates over dry ice, placed in individual air-tight plastic bags, and stored at −80°C until use. The other hemisphere was fixed by immersion in 4% buffered formalin for 3 weeks. The brain stem was cut on tangential sections 2 mm thick which were alternately frozen at −80°C or fixed in buffered formalin for 3 weeks. The neuropathological study for diagnosis was carried out with selected 4 μm-thick de-waxed paraffin sections of representative brain regions processed for immunohistochemistry as detailed elsewhere (Ferrer, 2014). Neuropathological diagnosis was based on the Braak and Braak stages of neurofibrillary tangle (NFT) pathology (Braak and Braak, 1991; Braak et al., 2006) and Thal phases of β-amyloid deposits (Thal et al., 2002). Cases with combined pathologies, excepting small blood vessel disease, and cases with metabolic syndrome, hypoxia, seizures, and long agonic state, were excluded. Since early stages of sporadic AD may show only NFT pathology without β-amyloid deposition Ferrer, 2012; Braak and Del Tredici, 2015), and these changes are similar to those seen in Primary age-related tauopathy (PART) (Crary et al., 2014; Duyckaerts et al., 2015), no attempt was made here to distinguish between early AD and PART, thus considering as pathological cases those containing NFTs grade according to Braak categorization stages. Cases were divided in two series. One series was used for DNA methylation studies; the LC of both sides was dissected from frozen samples. These cases were as follows: middle-aged cases (MA): *n* = 3, mean age: 50 ± 1 years; NFT pathology stages I-II, *n* = 4, age: 68 ± 6.3; NFT pathology stages III-IV, *n* = 7, age 85.1 ± 7.0; NFT pathology stages V-VI, *n* = 6, age: 76.3 ± 12.5 years. The second series was used for RT-qPCR after dissection of LC from frozen sections, and immunofluorescence and confocal microscopy carried out on serial sections containing the LC fixed in buffered formalin. These cases were the following: MA, *n* = 9, age: 51.0 ± 6.1; NFT pathology stages I-II, *n* = 9, age: 64.6 ± 5.7; and NFT pathology stages III-IV, *n* = 13, age: 78.1 ± 8.0. Cases in the second series were not affected by β-amyloid deposits, as phases 1 and 2 of Thal do not affect the LC.

Cases are summarized in Table 1. No differences in gender distribution were observed in these series. See comments below about age differences among groups.

**Table 1:** Summary of cases analyzed in the present series.

**Case**	**Age**	**Sex**	**Thal**	**Braak**	**PMD**	**RIN**	**RTqPCR**	**Methylation**
1	64	M	1	I	04 h 35 min	6.2	X	-
2	73	M	0	I	07 h 05 min	6.6	X	-
3	56	W	1	I	08 h 00 min	6.3	X	-
4	67	M	0	I	14 h 40 min	5.9	X	-
5	70	M	1	I	02 h 00 min	7.6	X	-
6	61	M	0	I	04 h 30 min	6.9	X	-
7	66	M	0	I	12 h 10 min	5.8	X	-
8	68	W	1	II	04 h 30 min	6.7	X	-
9	57	M	0	II	04 h 30 min	6.7	X	-
10	90	W	1	III	04 h 00 min	7.1	X	-
11	78	W	A	III	06 h 00 min	6.8	X	-
12	69	M	0	III	13 h 10 min	7.2	X	-
13	64	M	2	III	06 h 00 min	7.0	X	-
14	90	W	1	III	04 h 00 min	7.1	X	-
15	73	M	0	III	04 h 15 min	8.0	X	-
16	75	M	1	III	03 h 25 min	6.2	X	-
17	76	M	1	III	06 h 00 min	5.8	X	-
18	76	M	1	III	06 h 00 min	5.8	X	-
19	78	W	2	III	06 h 00 min	6.8	X	-
20	74	M	2	IV	04 h 45 min	6.2	X	-
21	84	M	2	IV	10 h 50 min	7.0	X	-
22	89	M	2	IV	03 h 20 min	7.0	X	-
23	44	M	0	0	06 h 40 min	6.7	X	-
24	52	M	0	0	03 h 00 min	6.8	X	-
25	52	M	0	0	04 h 40 min	7.6	X	-
26	52	W	0	0	05 h 45 min	6.4	X	-
27	41	M	0	0	11 h 35 min	5.9	X	-
28	60	W	0	0	11 h 30 min	5.8	X	-
29	59	M	0	0	08 h 30 min	6.6	X	-
30	51	W	0	0	04 h 00 min	5.9	X	-
31	48	W	0	0	14 h 30 min	6.1	X	-
32	64	M	0	I	08 h 00 min	-	-	X
33	68	M	0	I	10 h 55 min	-	-	X
34	77	M	1	I	06 h 55 min	-	-	X
35	63	M	0	I	02 h 45 min	-	-	X
36	79	W	2	III	03 h 40 min	-	-	X
37	82	W	2	III	03 h 05 min	-	-	X
38	81	M	1	III	05 h 50 min	-	-	X
39	90	W	1	IV	09 h 55 min	-	-	X
40	81	W	3	IV	05 h 00 min	-	-	X
41	99	W	2	IV	05 h 00 min	-	-	X
42	84	M	2	IV	12 h 15 min	-	-	X
43	74	W	2	V	05 h 30 min	-	-	X
44	95	M	3	V	03 h 00 min	-	-	X
45	81	W	3	V	05 h 15 min	-	-	X
46	75	M	3	V	11 h 30 min	-	-	X
47	77	M	3	V	16 h 00 min	-	-	X
48	56	W	4	VI	07 h 00 min	-	-	X
49	59	M	0	0	06 h 25 min	-	-	X
50	53	M	0	0	07 h 25 min	-	-	X
51	46	M	0	0	15 h 00 min	-	-	X

*Age in years; M, man; W, woman; Thal phases of β-amyloid deposition; Braak stages of neurofibrillary tangle pathology; PMD, post-mortem delay; RIN, RNA integrity number; RTqPCR, cases used for RT-qPCR and immunofluorescence; Methylation, cases assessed for DNA methylation. Cases 23-31 and 49–51 were considered middle-aged (MA) individuals with no NFT and plaque pathology*.

Regarding clinical phenotype, MA cases and cases with NFT pathology stages I-III were normal; some cases at stage IV (cases 20, 40 and 41) had suffered from mild cognitive impairment, and cases at stages V-VI were categorized as dementia of AD type.

### DNA extraction and Illumina Infinium Human MethylationEPIC BeadChip

Cases used for DNA methylation are detailed in Table 1. Total DNA was isolated from microdissected LC with DNeasy Blood and Tissue Kit (Qiagen, Madrid, Spain) according to the manufacturer's instructions. All DNA samples were assessed for integrity, quantity, and purity with electrophoresis in a 1.3% agarose gel, with picogreen quantification and nanodrop measurements. Bisulfite conversion of 500 ng of genomic DNA was performed using EZ DNA methylation kit (Zymo Research, Irvine, CA, USA) following the manufacturer's instructions. 200 ng of bisulfite-converted DNA was used for hybridization on the Illumina Infinium Human MethylationEPIC BeadChip (Illumina Inc., San Diego, CA, USA). Briefly, samples were whole genome-amplified, followed by an enzymatic end-point fragmentation, precipitation, and re-suspension. The re-suspended samples were hybridized onto the bead-chip for 16 h at 48°C and then washed. A single nucleotide extension with labeled dideoxy-nucleotides was performed, and repeated rounds of staining were applied with a combination of fluorescently labeled antibodies differentiating between biotin and DNP. Fluorescent signal from the microarray was measured with a HiScan scanner (Illumina Inc.,) using iScan Control Software (V 3.3.29). A three-step-based normalization procedure was performed using a package available for Bioconductor (Gentleman et al., 2004), under the R statistical environment, consisting of color bias adjustment, background level adjustment, and quantile normalization across arrays (Du et al., 2008). Methylation level (β-value) for each of the 866,836 CpG sites was calculated as the ratio of methylated signal divided by the sum of methylated and unmethylated signals plus 100. All beta values with an associated *p* ≥ 0.01 were removed from the analysis.

### Gene expression validation

Samples used for RT-qPCR analysis are detailed in Table 1. RNA from frozen dissected LC was extracted following the instructions of the supplier (RNeasy Mini Kit; Qiagen GmbH, Hilden, GE). RNA integrity number (RIN) and 28/18 S ratios were determined with the Agilent Bioanalyzer (Agilent Technologies, Santa Clara, CA, USA) to assess RNA quality; RNA concentration was evaluated using a NanoDropTM Spectrophotometer (Thermo Fisher Scientific, Carlsbad, CA, USA). RIN (RNA integrity number) values varied from 5.8 to 7.6 with no significant differences among groups. Retro-transcription reaction of RNA samples was carried out with the high-capacity cDNA archive kit (Applied Biosystems, Foster City, CA, USA) following the guidelines provided by the manufacturer and using Gene Amp 9700 PCR System thermocycler (Applied Biosystems). One sample of RNA was processed in parallel in the absence of reverse transcriptase to rule out DNA contamination.

Quantitative real-time polymerase chain reaction (RT-qPCR) assays were conducted in duplicate on cDNA samples obtained from the retro-transcription reaction diluted 1:10 in 384-well optical plates (Kisker Biotech, Steinfurt, GE) utilizing the ABI Prism 7900 HT Sequence Detection System (Applied Biosystems). TaqMan probes (Thermo Fisher Scientific) were Hs00390731_m1 (*KIAA0556* probe) and Hs01125994_m1 (*UTRN* probe). The mean value of four house-keeping genes, alanyl-transfer RNA synthase (AARS) (Hs00609836_m1), glucuronidase Beta (*GUS-*β) (Hs00939627_m1), hypoxanthine-guanine phosphoribosyltransferase (*HPRT1*) (Hs02800695_m1), and X-prolyl amino-peptidase (aminopeptidase P) 1 (*XPNPEP1*) (Hs00958026_m1), were used as internal controls for normalization of LC samples (Barrachina et al., 2006; Durrenberger et al., 2012). DDCT values were obtained from the DCT of each sample minus the mean DCT of the population of control samples (calibrator samples). The fold change was determined using the equation 2DDCT. Mean fold change values in each group were analyzed with the Students *t*-test using the Statgraphics Statistical Analysis and Data Visualization Software version 5.1.

### Double-labeling immunofluorescence and confocal microscopy

Cases used for immunofluorescence are named 10–31 in Table 1 (stages I-II were not examined). Three de-waxed sections, 4 μm thick, per selected cases were stained with a saturated solution of Sudan black B (Merck, Barcelona, Spain) for 15 min to block the auto-fluorescence of lipofuscin granules present in cell bodies, and then rinsed in 70% ethanol and washed in distilled water. The sections were incubated at 4°C overnight with combinations of primary antibodies: anti-KIAA0556 diluted 1:250 (NBP1-91006, NovusBiologicals, USA), anti-p60 katanin subunit diluted 1:250 (MAB7100, R&D systems, USA), anti-p80 katanin subunit diluted 1:150 (ab224171, Abcam, UK), and antibody AT8 diluted 1:50 (MN1020, ThermoFisher, USA). After washing, the sections were incubated with Alexa488 or Alexa546 (1:400; Molecular Probes, Eugene, OR, USA) fluorescence secondary antibodies against the corresponding host species. Nuclei were stained with DRAQ5^TM^ (1:2,000; Biostatus, Shepshed, UK). After washing, the sections were mounted in Immuno-Fluore mounting medium (ICN Biomedicals, Santa Clara, CA, USA), sealed, and dried overnight. Sections were examined with a Leica TCS-SL confocal microscope (Leica, Barcelona, Spain), and the images were acquired with Leica confocal software. Densitometry of the immunoreaction signal in LC positive cells for KIAA0556 and p80 was performed using Photoshop software in three different sections per case. Comparisons were made between control and diseased cases, and between neurons with and without NFT pathology in pathological cases. Densitometry of p60 immunostaining was performed in the whole neuropil of the LC due to punctate characteristics of p60 immunostaining.

### Statistical analysis

The effect of age and NFT pathology were determinant in DNA methylation and RT-qPCR expression. These aspects were further interpreted on the basis of immunohistochemical studies and quantification of data.

The normality of the data was assessed with the Shapiro-Wilk test or Kolmogorov–Smirnov test when required. DNA methylation data and RT-qPCR data were compared with one-way analysis of variance (ANOVA) followed by Tukey post-test. Statistical analysis and graphic design were performed with GraphPad Prism version 5.01 (La Jolla, CA, USA). Outliers were detected using the GraphPad software QuickCalcs (*p* < 0.05). Significance levels were set at ^*^p < 0.05 and ^**^p < 0.01. Statistical analysis of densitometric protein levels between groups, as revealed by immunofluorescence, was performed using t-student's test or one-way analysis of variance (ANOVA) followed by Tukey post-test when required using the SPSS software (IBM Corp. Released 2013, IBM SPSS Statistics for Windows, Version 21.0. Armonk, NY: IBM Corp.). Outliers were detected using the GraphPad software QuickCalcs (*p* < 0.05). All data were expressed as mean values ± SEM. Differences between middle-aged and NFT(+) were considered statistically significant at ^*^p < 0.05, ^**^p < 0.01, ^***^p < 0.001, and set at $ *p* < 0.05 and $$ *p* < 0.01 when comparing NFT(–) and NFT(+) neurons.

## Results

### Differential methylation regions

**Figure 1:**
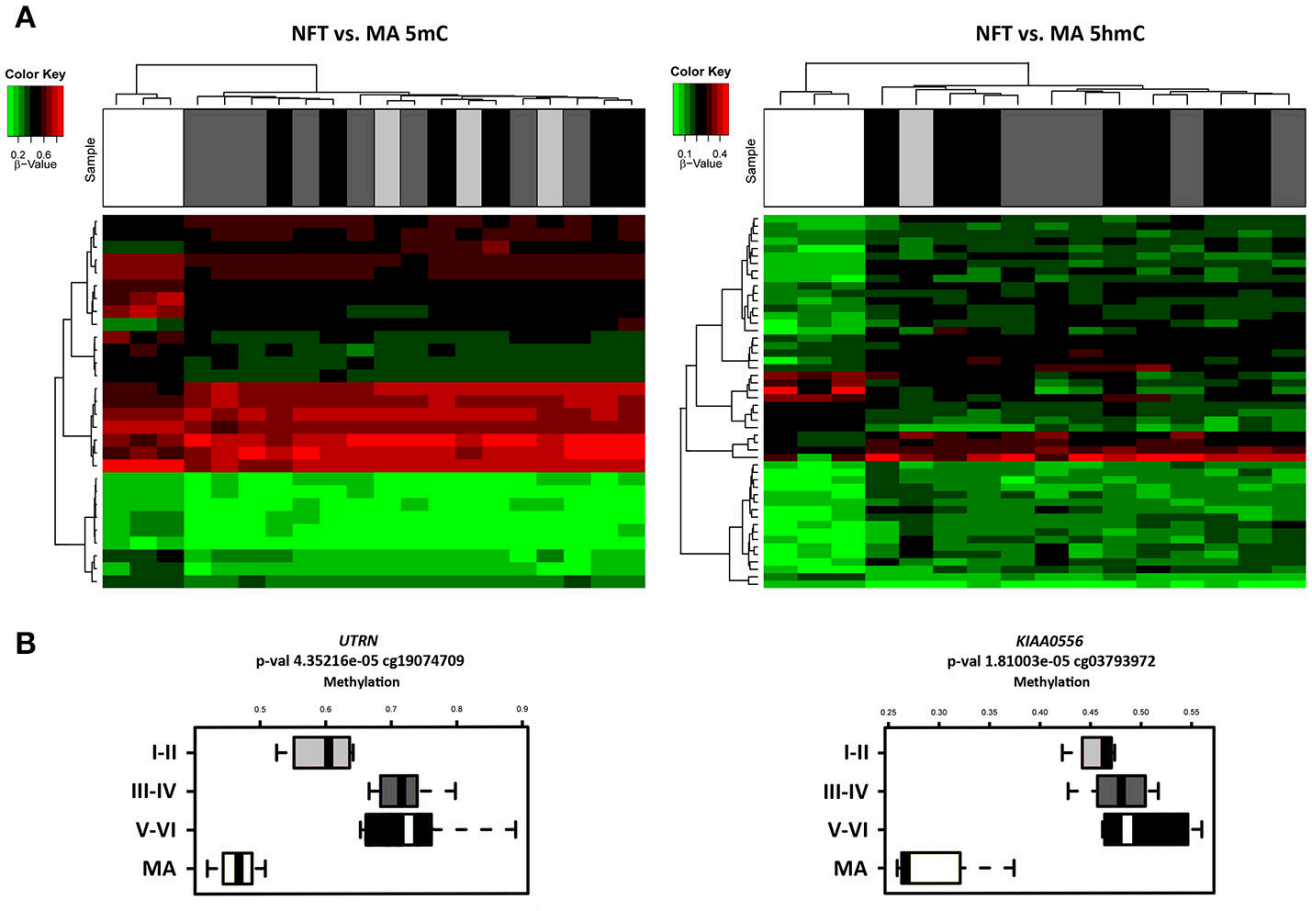
**(A)** Hierarchical clustering heat map of methylation (5mC) (left) and hydroxmethylation (5hmC) (right) array showing differential methylation profile in LC in cases with neurofibrillary tangle (NFT) pathology and middle-aged individuals (MA). Differences between groups are here considered statistically significant at an adjusted *p* < 0.05 in methylation study and at an unadjusted *p* < 0.05 in hydroxymethylation study. **(B)** Box plot of CpG methylation differences between MA and cases at different stages of NFT pathology (I-II, III-IV, and V-VI) in *UTRN* (left) and *KIAA0556* (right) genes. Significant differences in *KIAA0556* DNA 5mC are seen between MA and all stages of NFT pathology. Abbreviations: 5mC: methylation; 5hmC: hydroxymethylation, *UTRN*: utrophin; *KIAA0556*: Katanin-interacting protein. Samples color code: gray: MA cases; black: cases with NFT pathology.

Illumnia Infinium Human MethylationEPIC BeadChip Kit was used, covering over 850,000 methylation sites quantitatively across the genome at single-nucleotide resolution, which permits discrimination between 5mC and 5hmC. DNA methylation profiles obtained from this platform showed a few differential methylation regions (DMRs) when comparing MA individuals with cases showing various stages of NFT pathology. After the *Lumi* software analysis, coding DMRs showing significant differences at an adjusted *p* < 0.05 were sorted and ranked according to their CpG mean differences and significance (Supplementary Table 1). In contrast, 5hmC data obtained when comparing NFT stages with MA cases did not reveal significant differences following the same adjusted *p*-value established for 5mC (Figure 1A). For this reason no further attempt was made to analyze gene expression of suspected hydroximethylated genes. Enrichment analysis against the “Go Ontology” database did not identify significant clusters for coding DMRs in ases with NFT pathology. Two significant DMRs, *UTRN* and *KIAA0556* genes, coding for utrophin and katanin-Interacting Protein, respectively, were differentially hypo-methylated in cases with NFT pathology when compared with MA individuals. Differences were more marked for *KIAA0556* when comparing MA with early stages of NFT pathology (Figure 1B).

### Gene expression validation

**Figure 2:**
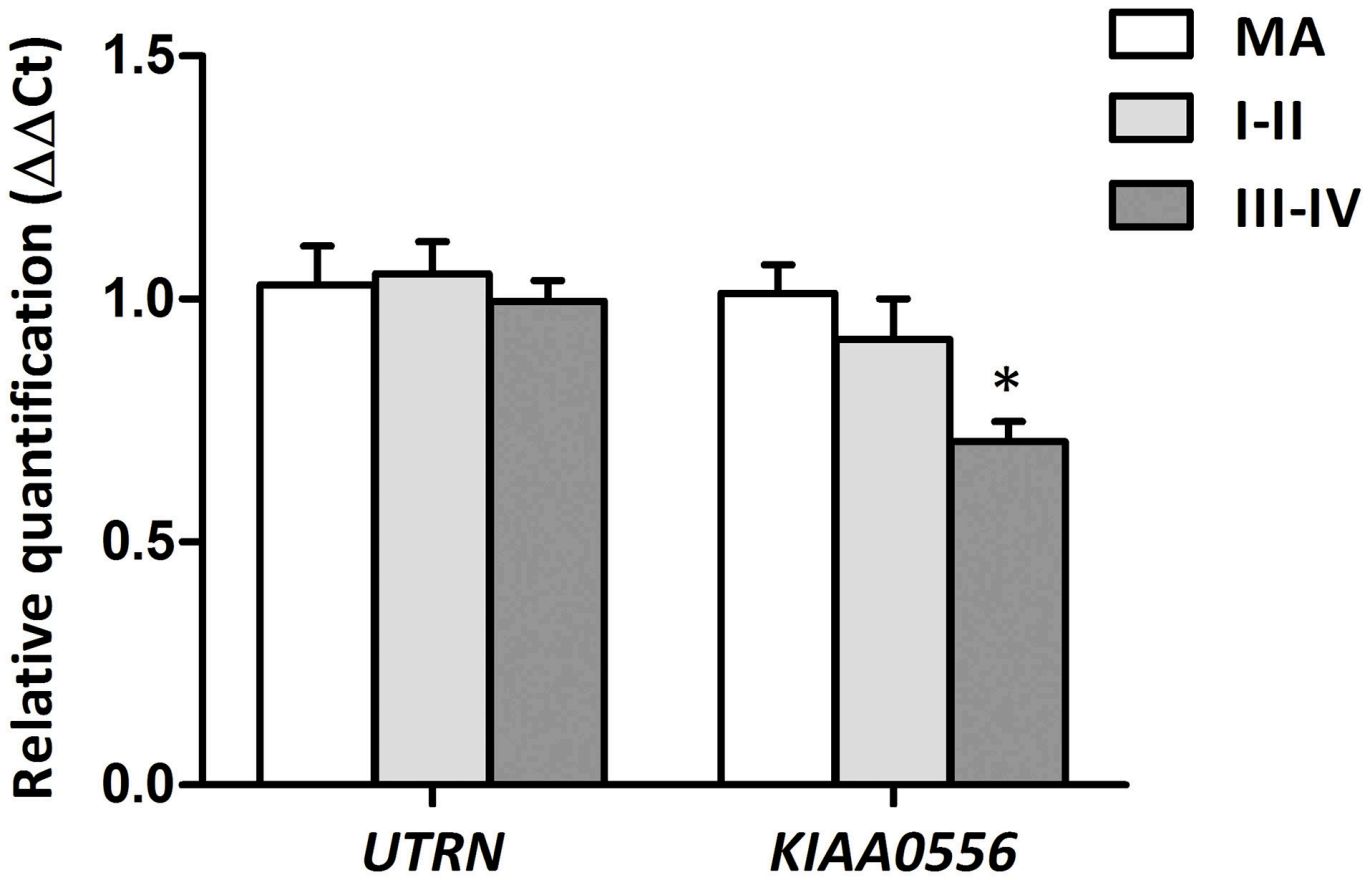
Gene expression levels of *UTRN* and *KIAA0556* genes in LC in MA and cases with NFT pathology at stages I-II and III-IV. *p < 0.05 when comparing MA with NFT III-IV.

RT-qPCR was performed to evaluate *UTRN* and *KIAA0556* mRNA expression. *UTRN* mRNA expression did not significantly differ in MA and cases with NFT pathology. In contrast, *KIAA0556* mRNA expression was significantly reduced at stages III-IV of NFT pathology when compared with MA individuals (*p* = 0.002; Figure 2).

### Protein expression of KIAA0556, and katanin p80 and p60 subunits in LC

Double-labeling immunofluorescence and confocal microscopy to selected proteins and hyperphosphorylated tau (antibody AT8) were used to a: identify localization of KIAA0556, and katanin p80 and p60 subunits in LC; b: assess modified expression levels of these proteins in cases with NFT pathology; and c: analyze the relationship, if any, between KIAA0556, and katanin p80 and p60, in relation to tau deposition in neurons and neuropil of the LC.

**Figure 3:**
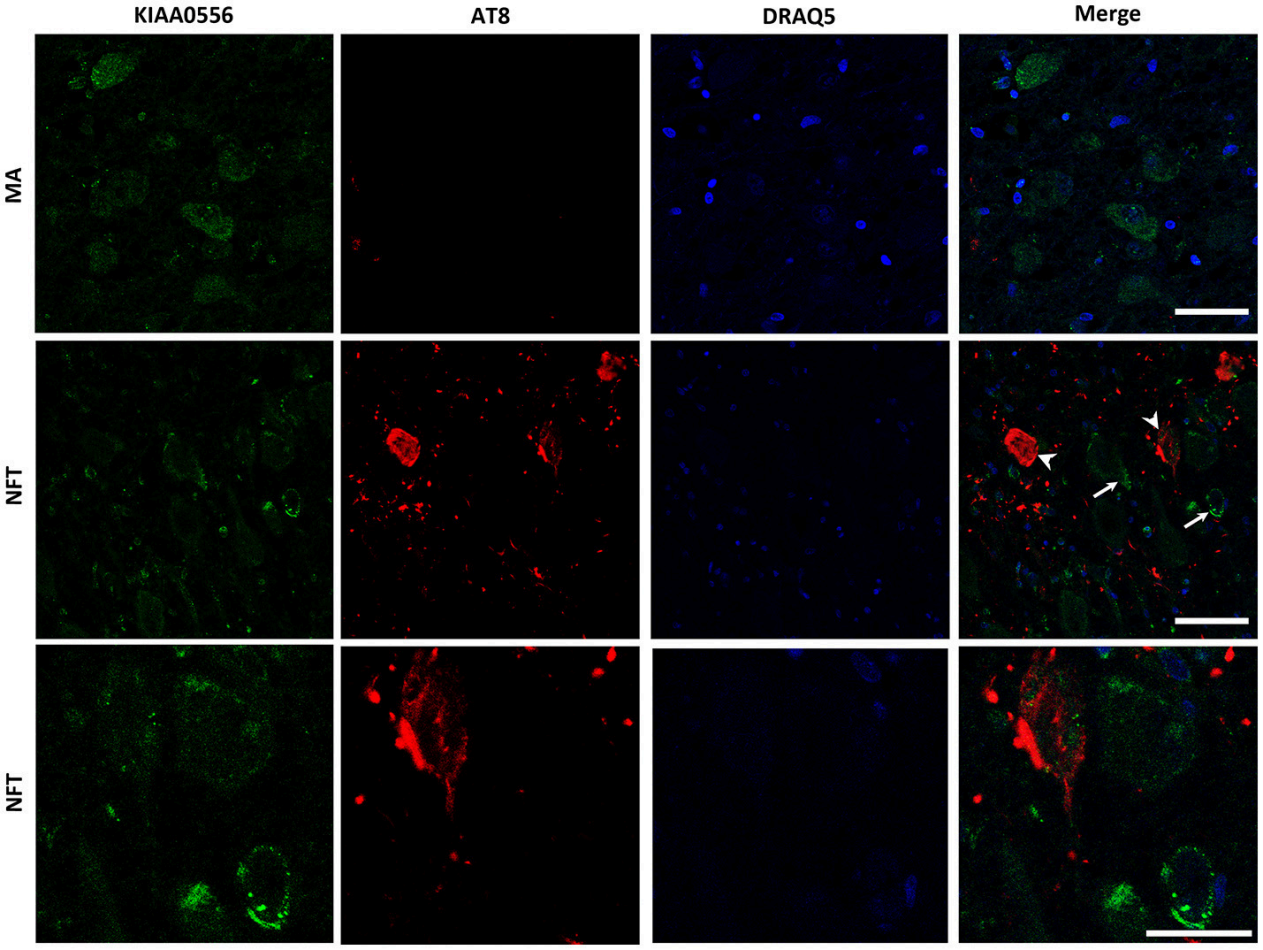
Double-labeling immunofluorescence and confocal microscopy to KIAA0556 (green) and hyper-phosphorylated tau (clone AT8: red) in MA and in one case with NFT pathology stage IV. Note decreased KIAA0556 restricted to neurons containing NFT (arrowhead: only red; arrows: only green). Lower row: higher magnification of the upper right corner showing decreased KIAA0556 immunoreactivity in one neuron containing NFT. Paraffin sections; nuclei stained with DRAQ5TM (blue); bar in the two upper rows = 50 μm; bar in the lower row = 25 μm.

KIAA0556 immunoreactivity was observed in the cytoplasm of neurons in MA and in cases with NFT pathology; the immunoreactivity was similar in neurons from MA cases and in neurons without NFTs in cases with NFT pathology. But KIAA0556 immunoreactivity was markedly reduced in neurons with NFTs, as revealed by double-labeling immunofluorescence and confocal microscopy (Figure 3). Quantitative studies showed no differences in the amount of protein in neurons of the LC in MA individuals and in non-containing NFT neurons in cases with NFT pathology stages III-IV. However, significant reduction of KIAA0556 immunoreactivity was verified in neurons containing hyper-phosphorylated tau deposits when compared with neurons not bearing NFTs in the same tissue section (*p* = 0.002) (Figure **6A**).

**Figure 4:**
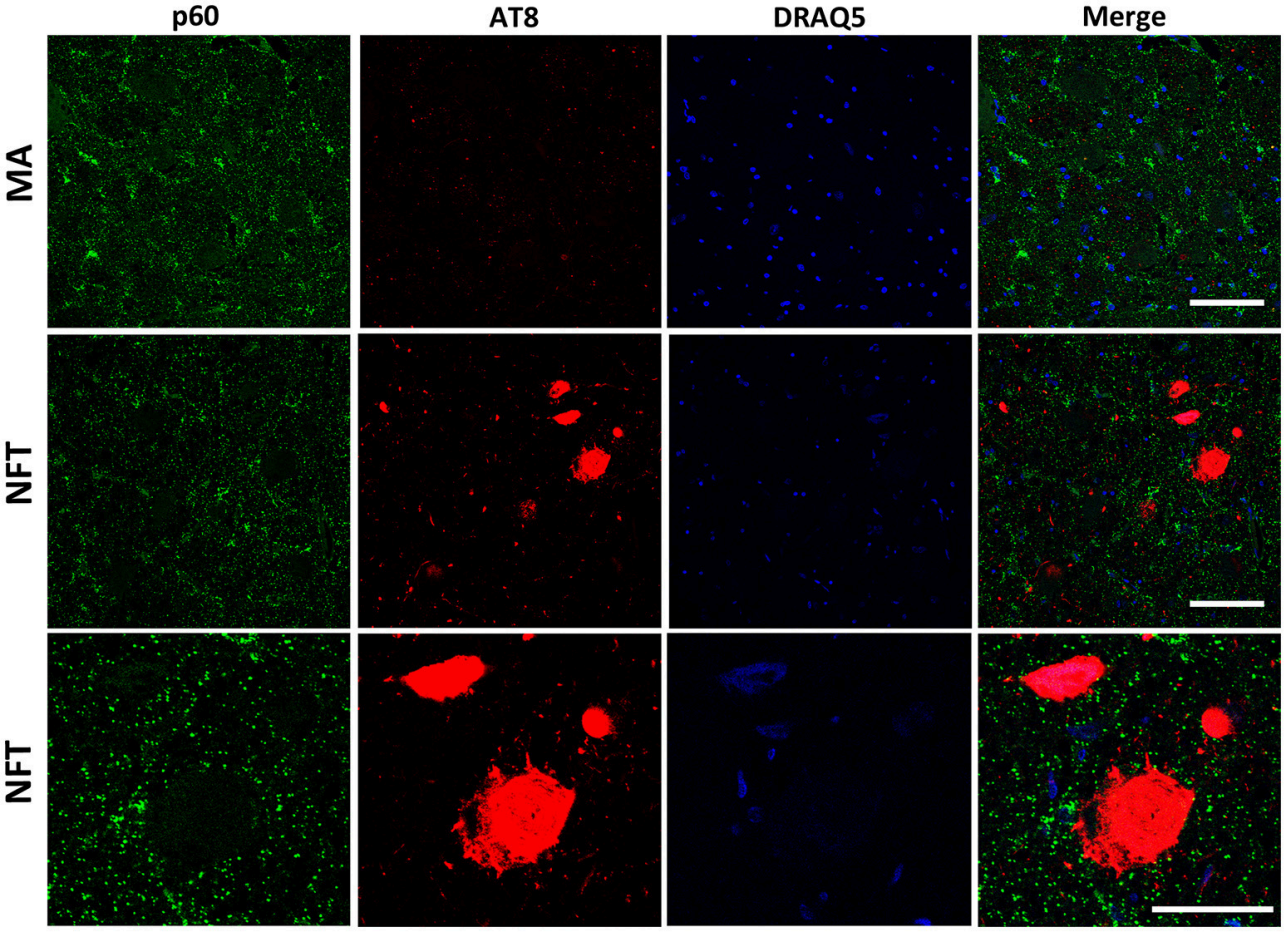
Double-labeling immunofluorescence and confocal microscopy to p60-katanin (green) and hyper-phosphorylated tau (clone AT8: red) in MA and in one case with NFT pathology stage IV. Lower row: higher magnification of the upper right corner showing preserved p60-katanin immunoreactivity in the neuropil in NFT case when compared with MA. Paraffin sections; nuclei stained with DRAQ5TM (blue); bar in the two upper rows = 50 μm; bar in the lower row = 25 μm.

To further learn about KIAA0556-associated proteins, double-labeling immunofluorescence to p60- and p80-katanin subunits was assessed. p60-katanin immunoreactivity was localized as punctuate, synaptic-like deposits in the neuropil in MA and cases at stages III-IV of NFT pathology, which was consistent with the localization of this protein in distal neuronal processes. Double-staining of p60-katanin and AT8 showed no apparent decrease in p60-katanin immunoreactivity in the neuropil of the LC in cases with NFT pathology when compared with MA (Figure 4). Densitometry revealed no significant differences between the two groups (*p* = 0.62; Figure **6B**).

**Figure 5:**
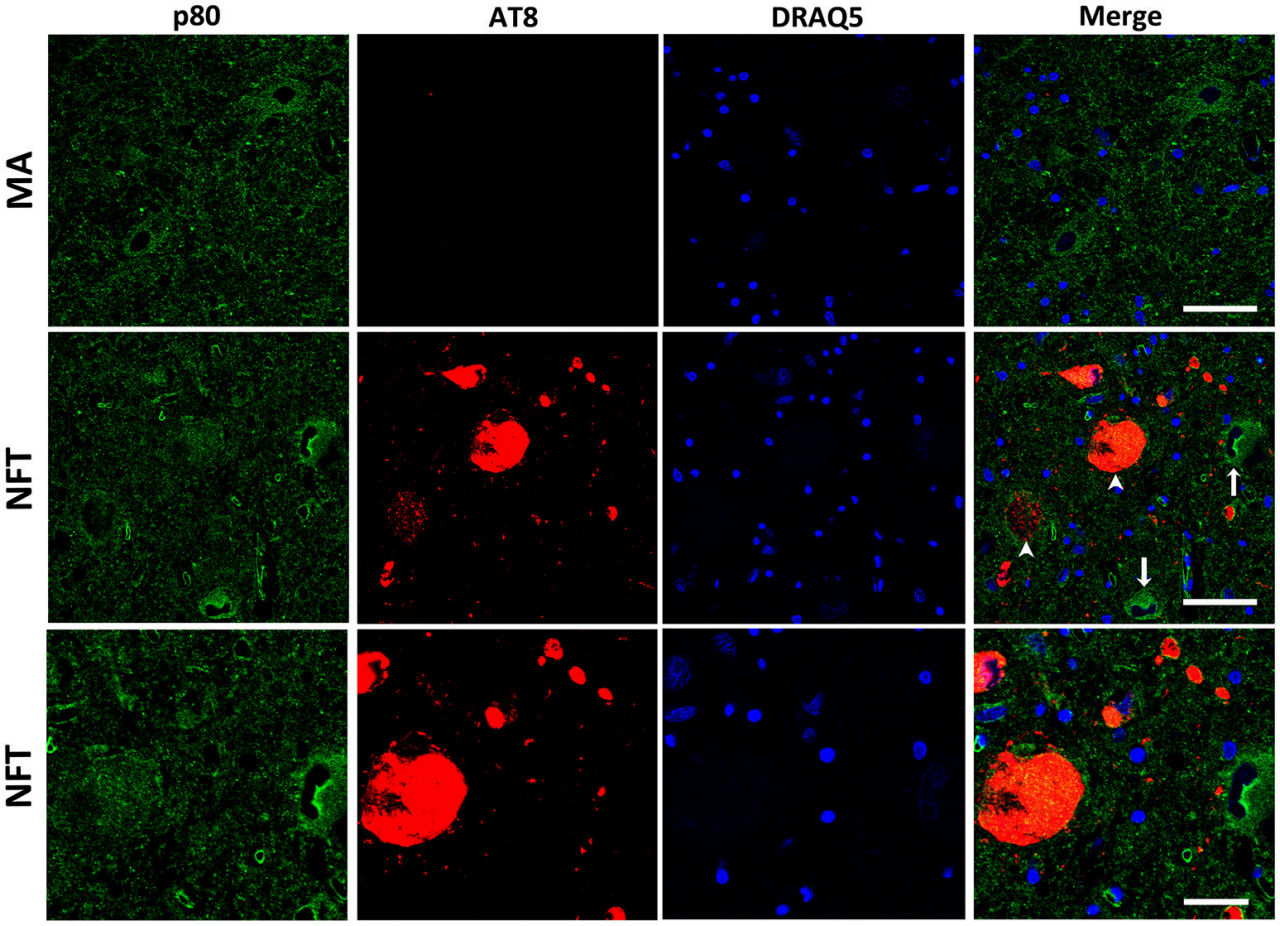
Double-labeling immunofluorescence and confocal microscopy to p80-katanin (green) and hyper-phosphorylated tau (clone AT8: red) in MA and in one case with NFT pathology stage IV. Note decreased p80-katanin restricted to neurons containing NFT (arrowhead: only red; arrows: only green). Lower row: higher magnification of the upper right corner showing decreased p80-katanin immunoreactivity in one neuron containing NFT. Paraffin sections; nuclei stained with DRAQ5TM (blue); bar in the two upper rows = 50 μm; bar in the lower row = 25 μm.

**Figure 6:**
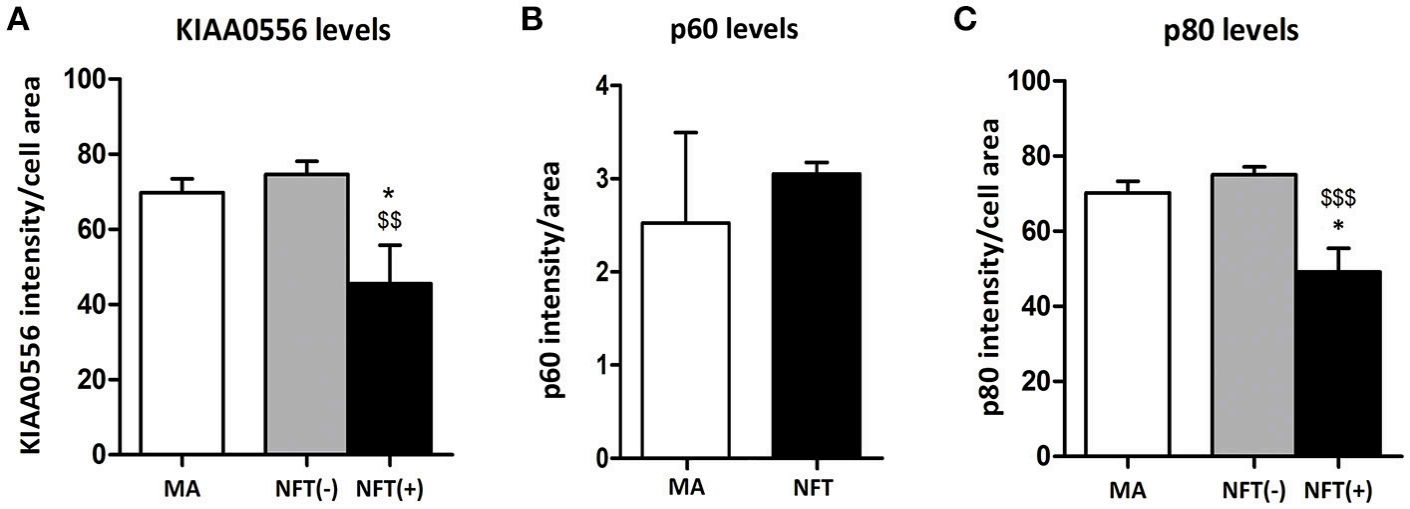
Densitometric study showing no differences in p60 protein expression between LC neurons in MA cases and tau non-bearing neurons in cases with NFT pathology **(B)**, but significant KIAA0556 **(A)**, and p80-katanin (p80) **(C)** reduction in NFT-containing neurons when compared with non-tau containing neurons in the same sections. *p < 0.05 when comparing MA with NFT, and $$*p* < 0.01 and $$$*p* < 0.001 when comparing NFT(−) and NFT(+) neurons in cases with NFT pathology.

Finally, p80-katanin protein expression was localized in the cytoplasm of LC neurons. p80-katanin immunoreactivity was selectively reduced in neurons of the LC containing hyperphosphorylated tau deposits (Figure 5). Densitometric analysis further demonstrated no differences between MA neurons and neurons without tau deposits in cases with NFT pathology, but significant reduction of p80-katanin immunoreactivity in neurons with NFTs when compared with neurons without NFTs in the same tissue section (*p* = 0.000; Figure 6C).

## Discussion

DNA methylation at 5-methylcytosine (5mC) is an epigenetic mechanism associated primarily with transcriptional repression, whereas methylation at 5-hydroxymethylcytosine (5hmC) has opposite effects. Here we used hybridization on the Illumina Infinium Human MethylationEPIC BeadChip to identify differences in DNA methylation of dissected LC of cases with NFT pathology compared with MA individuals without NFTs. The age of the two groups was different, with cases with NFT pathology being older than MA individuals. This is not a rare situation as 85% of human beings aged 65 years and older show NFT pathology defined, minimally, as stages I-II of Braak in the entorhinal cortex (EC). Most of them, if not all, have hyper-phosphorylated tau deposits in the LC (Braak et al., 2011; Ferrer, 2012). Indeed, tau pathology in certain nuclei of the brain stem, including LC, precedes tau pathology in the EC (Rüb et al., 2001; Grinberg et al., 2009; Simic et al., 2009; Attems et al., 2012). For this reason, the MA group was selected on the basis of the absence of tau pathology in LC. It can be argued that the present design is biased by age differences between the two main groups. This objection may be true in other situations, but present observations geared to learning about the association of NFT pathology and abnormal gene expression in neurons of the LC proved it not to be here.

Using restrictive conditions, two DNA hypo-methylated genes were selected for further study; *UTRN* and *KIAA0556* genes, coding for utrophin and katanin-Interacting Protein, were differentially hypo-methylated in cases with NFT pathology when compared with MA individuals. RT-qPCR showed no differences in *UTRN* mRNA expression between the two groups. However, *KIAA0556* mRNA expression was significantly reduced in cases with NFT pathology when compared with MA. Lack of correlation between degree of DNA methylation of CpG islands as revealed by the bisulphate methods and gene transcription is not a rare phenomenon in diseases of the nervous system (Nicolia et al., 2017).

Altered *KIAA0556* mRNA expression in neurons with NFT may have implications in microtubule pathology in neurons bearing hyper-phosphorylated tau in LC. Microtubules are hollow polymers of α- and β-tubulin subunits with one end, the plus-end, favored by the addition and subtraction of subunits, while the minus-end has limited turnover capacity; β-tubulins predominate at the plus-end and α-tubulins at the minus-end (Baas and Lin, 2011; Baas, 2013; Kapitein and Hoogenaad, 2015; Baas et al., 2016). This structure permits high plasticity; long microtubules shape the morphology and stability of dendrites and axons, and serve as a means to transport distinct molecules over long distances; short microtubules permit the growth of microtubules and the transport of tubulins (Baas et al., 2016). Stability of microtubules is in part related to tubulin polyamination catalyzed by transglutaminases (Song et al., 2013; Song and Brady, 2015). Several proteins bind to microtubules and modulate stabilization of microtubules; tau and other microtubule-associated proteins (MAPs) have repeats of microtubule-binding domains and prevent de-polymerization of microtubules (Mandelkow and Mandelkow, 1995; Kadavath et al., 2015). Tau hyper-phosphorylation, as occurs in NFTs, results in tau dissociation from microtubules and aberrant formation of paired helical filaments (Alonso et al., 1999; Buée and Delacourte, 2001; Avila, 2006; Goedert et al., 2006; Wang and Liu, 2008; Duan et al., 2012; Matamoros and Bass, 2016).

Microtubule-severing proteins are proteins which form hexamers on the surface of microtubules and break the microtubules into fragments that can be transported to distinct places of the cytoplasm, axon, and dendrites to produce new microtubules, as only short microtubules are able to be transported (Wang and Brown, 2002; Baas et al., 2006; Roll-Mecak and McNally, 2010). Katanin is one of the most abundant microtubule-severing proteins in brain and plays an important role in axonal growth and dendrite branching during development due to its participation in the generation of microtubules (Ahmad et al., 1999; Yu et al., 2005, 2008; Roll-Mecak and Vale, 2006). Increased activity of microtubule-severing proteins also has noxious effects as it is accompanied by degradation of the neuronal cytoskeleton (Yu et al., 2005; Sudo and Baas, 2011). p60-katanin is localized in neuronal processes and severs microtubules whereas p80-katanin is localized in the cytoplasm at the centrosomes (McNally et al., 2000; Yu et al., 2005). Tau seems to protect microtubules in the axon from severing by katanin (Qiang et al., 2006). Following on from this posit, it has been suggested that hyper-phosphorylated tau loses its function as protector and randomly enables microtubule severing by katanin (Qiang et al., 2006; Sudo and Baas, 2011).

KIAA0556 co-localizes with α-tubulin, and binds to p60- and p80-katanin subunits (Sanders et al., 2015). Moreover, KIAA0556 seems to negatively regulate katanin severing (Sanders et al., 2015). Mutations of KIAA0556 gene are causative of Joubert syndrome, which is manifested by several malformations including brain (Sanders et al., 2015; Roosing et al., 2016).

Considering all these data, the expression of proteins KIAA0556, and p60- and p80-katanin was considered a next step in the study of possible alterations of this pathway in the LC in neurons with NFT pathology. Double-labeling immunofluorescence with antibodies to these proteins, and to phosphorylated tau, disclosed no differences in the expression of KIAA0556 and p80-katanin in LC neurons of MA cases and in neurons without hyper-phosphorylated tau deposits in cases with NFT pathology. Moreover, no significant differences were seen in p60-katenin expression in the neuropil of cases with and without NFT pathology. These findings support the idea that age, *per se*, is not a determining factor in the altered expression of KIAA0556 and p80-katanin in LC neurons. Yet it is the presence of NFTs which makes the difference; KIAA0556 and p80-katanin protein are reduced only in neurons with NFTs.

Together, the present findings show association of decreased KIAA0556 and p80-katanin subunits in LC neurons with NFTs, and suggest that altered microtubule homeostasis in those neurons is linked to deregulation of the katanin-signaling pathway.

## Author contributions

PA-B: RT-qPCR, immunohistochemistry; RD-M: DNA methylation studies; IF: Design of the study, sample selection, data interpretation, and writing of the manuscript.

### Conflict of interest statement

The authors declare that the research was conducted in the absence of any commercial or financial relationships that could be construed as a potential conflict of interest.
