# Supplementary material for: Altered Regulation of KIAA0566, and Katanin Signaling Expression in the Locus Coeruleus With Neurofibrillary Tangle Pathology
Source: Front Cell Neurosci. 2018 May 17;12:131. doi: 10.3389/fncel.2018.00131 (PMC5966574; doi:10.3389/fncel.2018.00131)
Supplement: Supplementary file 1 [file Supplementary_Table_1.docx]

**Supplementary table 1:** Top 30 differently methylated regions (5mC) in LC in Alzheimer's disease. Significance is set at an adjusted p-value < 0.05. Abbreviations: Chr: chromosome; FC: Fold Change; MA: middle-aged individuals; AD: Alzheimer's disease. Note that values are expressed comparing MA vs AD; therefore *KIAA0556* is hypo-methylated in AD when compared with MA.

| **Methylated regions** | | | | | | |
| --- | --- | --- | --- | --- | --- | --- |
| **Region** | **Gene symbol** | **Chr** | **Gene name** | **LogFC MA vs AD** | **Probe ID** | **Adj. p-value** |
| Region_01 | *GPX5* | 6 | Glutathione Peroxidase 5 | -0.21 | cg18361418 | 0.037 |
| Region_02 | *ATP6V0A4* | 7 | ATPase H+ Transporting V0 Subunit A4 | -0.18 | cg03793972 | 0.037 |
| Region_03 | *CALCB* | 11 | Calcitonin Related Polypeptide Beta | -0.16 | cg23721036 | 0.037 |
| Region_04 | *-* | 6 | - | -0.15 | cg08125215 | 0.045 |
| Region_05 | *-* | 6 | - | -0.14 | cg15938671 | 0.037 |
| Region_06 | *-* | 5 | - | -0.14 | cg04991179 | 0.049 |
| Region_07 | *LOC100287792* | 20 | Uncharacterized LOC100287792 | -0.13 | cg15270427 | 0.016 |
| Region_08 | *-* | 6 | - | -0.10 | cg26164878 | 0.049 |
| Region_09 | *CYP3A43* | 7 | Cytochrome P450 Family 3 Subfamily A Member 43 | -0.09 | cg21542643 | 0.049 |
| Region_10 | *HOXA-AS3* | 7 | HOXA Cluster Antisense RNA 3 | 0.06 | cg10343278 | 0.037 |
| Region_11 | *TMC2* | 20 | Transmembrane Channel Like 2 | 0.06 | cg25182418 | 0.049 |
| Region_12 | *-* | 4 | - | 0.07 | cg11722587 | 0.049 |
| Region_13 | *MFHAS1* | 8 | Malignant Fibrous Histiocytoma Amplified Sequence 1 | 0.08 | cg20497631 | 0.045 |
| Region_14 | *C10orf46* | 10 | CDK2 Associated Cullin Domain 1 | 0.08 | cg27009448 | 0.037 |
| Region_15 | *-* | 8 | - | 0.08 | cg20601772 | 0.037 |
| Region_16 | *-* | 3 | - | 0.09 | cg06320828 | 0.037 |
| Region_17 | *UBXN10* | 1 | UBX Domain Protein 10 | 0.10 | cg18722282 | 0.045 |
| Region_18 | *AFF3* | 2 | AF4/FMR2 Family Member 3 | 0.11 | cg14666720 | 0.037 |
| Region_19 | *CLCNKB* | 1 | Chloride Voltage-Gated Channel Kb | 0.12 | cg03249707 | 0.037 |
| Region_20 | *DDAH1* | 1 | Dimethylarginine Dimethylaminohydrolase 1 | 0.13 | cg06514399 | 0.037 |
| Region_21 | *-* | 5 | - | 0.13 | cg02640809 | 0.037 |
| Region_22 | *GSC* | 14 | Goosecoid Homeobox | 0.13 | cg23074992 | 0.020 |
| Region_23 | *-* | 2 | - | 0.15 | cg22344631 | 0.037 |
| Region_24 | *CDYL* | 6 | Chromodomain Y Like | 0.15 | cg18559739 | 0.037 |
| Region_25 | *LHX2* | 9 | LIM Homeobox 2 | 0.15 | cg14093715 | 0.037 |
| Region_26 | *SCNN1A* | 12 | Sodium Channel Epithelial 1 Alpha Subunit | 0.18 | cg04894439 | 0.037 |
| Region_27 | *VAX2* | 2 | Ventral Anterior Homeobox 2 | 0.19 | cg17649293 | 0.037 |
| Region_28 | *UTRN* | 6 | Utrophin | 0.20 | cg19224759 | 0.028 |
| Region_29 | *-* | 6 | - | 0.20 | cg15342087 | 0.016 |
| Region_30 | *KIAA0556* | 16 | Katanin-Interacting Protein | 0.26 | cg02113604 | 0.045 |
